# Climate Mobility and Development Cooperation

**DOI:** 10.1007/s11111-021-00387-5

**Published:** 2021-07-17

**Authors:** Robert Stojanov, Sarah Rosengaertner, Alex de Sherbinin, Raphael Nawrotzki

**Affiliations:** 1 Faculty of Business and Economics, Mendel University in Brno, Zemedelska 1, 613 00 Brno, Czech Republic; 2 The New School, Zolberg Institute On Migration and Mobility, NY, USA; 3 Center for International Earth Science Information Network (CIESIN), The Earth Institute, Columbia University, NY, USA; 4 German Institute for Development Evaluation (Deval), Bonn, Germany

## Abstract

Development cooperation actors have been addressing climate change as a cross-cutting issue and investing in climate adaptation projects since the early 2000s. More recently, as concern has risen about the potential impacts of climate variability and change on human mobility, development cooperation actors have begun to design projects that intentionally address the drivers of migration, including climate impacts on livelihoods. However, to date, we know little about the development cooperation’s role and function in responding to climate related mobility and migration. As such, the main aim of this paper is to outline the policy frameworks and approaches shaping development cooperation actors’ engagement and to identify areas for further exploration and investment. First, we frame the concept of climate mobility and migration and discuss some applicable policy frameworks that govern the issue from various perspectives; secondly, we review the toolbox of approaches that development cooperation actors bring to climate mobility; and third, we discuss the implications of the current Covid-19 pandemic and identify avenues for the way forward. We conclude that ensuring safe and orderly mobility and the decent reception and long-term inclusion of migrants and displaced persons under conditions of more severe climate hazards, and in the context of rising nationalism and xenophobia, poses significant challenges. Integrated approaches across multiple policy sectors and levels of governance are needed. In addition to resources, development cooperation actors can bring data to help empower the most affected communities and regions and leverage their convening power to foster more coordinated approaches within and across countries.

## Introduction

The literature on climate mobility largely agrees that climate factors contribute to migration (both voluntary and forced), but that their contribution generally operates through socioeconomic factors such as wage differentials, family reunification, and the quest for improved living standards (Foresight, 2011). Controlling for other factors known to influence migration decision making, researchers have attributed migration to deviations in temperature and precipitation (Nawrotzki et al., 2015b) and to climate extremes such as storms, floods, and drought (Berlemann & Steinhardt, 2017). Many of these impacts are traced through the “agricultural pathway” (Nawrotzki & Bakhtsiyarava, 2017), meaning that the impact of climate on migration is moderated by changes in agricultural productivity.

In the first decade of the twenty-first century, climate adaptation became part of the development cooperation agenda, and aid agencies increasingly focused on mainstreaming adaptation into their development portfolios (Eriksen & Naess, 2003, Huq & Reid, 2004; Agrawala & Van Aalst, 2008). Today, development cooperation actors—defined here as encompassing donors of development assistance (including governments acting through bilateral and multilateral channels, as well as non-governmental and private donors) and implementing agencies that provide technical assistance^1^—have come to realize that climate information related to past variability, current change, and future projections is vital for understanding how various sectors may be stressed by climate extremes and at risk of future impacts. Yet, while we now have a better understanding of the current and projected impacts of climate change on various economic sectors, the social and political dimensions of population responses to climate change remain a topic of active research (IPCC, 2014, 2018).

Migration is an integral part of development processes (UNCTAD, 2018; UNDP, 2009). Yet, the development policy discourse has long conceived of migration as a symptom of development failure, as evidenced in a recurring focus on addressing the “root causes” of migration (e.g., ICPD, 1994; UNGA, 2018a). International policy discussions on migration have traditionally focused on risks—such as migration draining developing countries of their “best and brightest” (Özden & Schiff, 2006)—and vulnerabilities, including the exploitation of migrant workers and protection needs of refugees and internally displaced persons. At the beginning of the 2000s, a more positive discourse emerged (GCIM, 2005; UNGA, 2006, 2013), based on evidence that the size of global remittances far outstrips official development assistance (ODA) (e.g., Ratha & Xu, 2008), and of migration’s tangible contributions to poverty reduction (e.g., Ratha, 2013) and human development (UNDP, 2009). However, with the bifurcation of aid flows and international institutions into humanitarian and development mandates, the needs of migrants and refugees in developing countries have traditionally been addressed by humanitarian actors. This changed as the mass exodus of refugees from Syria, which overwhelmed neighboring countries and led to a spike in arrivals to Europe in 2015, ushered in recognition that greater global responsibility-sharing is needed (UNGA, 2016a, b, 2018b). Donors, international agencies, and non-governmental organizations pledged to better align humanitarian and development assistance (UNGA, 2016a). Consequently, development cooperation actors have been drawn into responding to large movements and protracted refugee situations in developing countries. The case for a developmental approach to displacement has been further bolstered by an increasing body of research making the case for the benefits of fostering refugee self-reliance and integration in local labor markets and economies (e.g. Betts et al., 2016).

As development cooperation actors have become increasingly active in addressing migration and displacement situations, this paper seeks to shed light on their role in anticipating and responding to climate-related mobility. As such, the goal of this paper is to explore the existing “toolbox” that development cooperation actors bring to this issue and to identify areas for further exploration and activity. We do not seek to settle the sometimes controversial question of whether the effects of continued global warming will trigger large-scale movements. Large movements are already occurring, in some cases with climate anomalies as antecedents (Kelley et al., 2015), and development cooperation actors have been called upon to respond. We examine the tools and approaches they have developed and how those might need to be adapted to address the challenge of climate related mobility.

We focus on human mobility as a broad concept encompassing various types of movement including voluntary migration as adaptation, forced displacement, and planned relocation of communities. We examine how development cooperation actors approach the nexus of climate change adaptation and migration. We chose this approach to cover the depth and breadth of development cooperation actors’ responses to climate-related mobility. However, for specific cases, we clearly reference the particular type of mobility under discussion.

This review article is based on a comprehensive review of more than 25 reports produced by development agencies on the subject of climate mobility, and a review of 40 articles or reports on development interventions and programmes related to climate-related migration or associated issues of climate hazard displacement. It also reflects the more than 50-year collective experience of the authors in the field of climate related mobility, as well as engagement with development cooperation actors such as the World Bank, US Agency for International Development (USAID), United Nations Development Programme (UNDP), and Environment Programme (UNEP) on issues of climate vulnerability, adaptation, and migration. Rather than a systematic literature review, this article presents a policy-oriented discussion of how the development community has approached the complex issues around climate mobility and what might be done in the future.

We divide our exposition in three sections. “Framing climate mobility: concepts and policy frameworks” defines the frameworks of climate change and migration or mobility and discusses migration as a response to climate hazards and how various policy frameworks treat the issue, “The role of development cooperation in addressing climate mobility” explores how development cooperation actors approach climate mobility, and “Future prospects and policy implications” highlights challenges and opportunities for the way forward.

### Framing climate mobility: concepts and policy frameworks

Environmental migration is generally understood as a form of forced migration (Hugo, 1996) in which environmental changes, generally operating through economic, social, and political factors (Foresight, 2011), induce people to move outside their original habitats. The concept of climate migration is narrower, and often used interchangeably with other terms such as climate-induced migration, climate displacement, climate refugee flows, climate-related movement, and climate mobility. There is no consensus or definitional clarity about what terminology to use (Ferris, 2020; McMichael et al., 2021). Terms like climate refugee are popular but problematic (de Sherbinin, 2020), since refugee status is conferred based on international law and limited to people crossing an international border and fleeing persecution owing to strictly define factors such as their race, ethnicity, faith, membership of a particular social group, or political beliefs.^2^ Scientists are increasingly adopting the term climate mobility (Baldwin et al., 2019; Farbotko, 2020), which acknowledges the diversity of forms and directions of population movement in the context of climate variability and change (Boas et al., 2019).

People can respond to climate stressors by either adapting in place, for instance, through livelihood changes or diversification, or they may decide to move elsewhere (Nawrotzki et al., 2015a). In some cases, they may pursue a mixed strategy with some members of the household moving, while others stay behind, leading to translocal and transnational ties that sustain households (Greiner et al., 2015). Movement responses are shaped by the sudden or slow-onset nature of climate-related impacts: Fast-onset events (floods, cyclones) tend to trigger reactive movements of a more temporary nature (people return when the event subsides), whereas slow-onset events (such as sea-level rise or increases in temperature) may be associated with more anticipatory and durable patterns of mobility, whereby return is less likely if not impossible (Kalin, 2010, Black et al., 2011, Bohra-Mishra et al., 2014; Nawrotzki et al., 2016). However, repeated climate-related disasters may either erode household assets to the point that people are “trapped” in place (Ayeb Karlsson et al., 2018, Black et al., 2011) or they determine that is best to move on (Rigaud et al., 2018).

Newer research seeks to deepen our understanding of the inherent propensity of certain subpopulations to consider migration as an option in the face of climate and other risks (Adams & Kay, 2019). Furthermore, many people do not wish or perceive the need to move despite increasing climate risks or declines in habitability (Adger et al., 2021). It remains a consistent finding that even in areas with apparently declining climatic and environmental conditions, migrants, when interviewed, still primarily cite economic motivations for moving (Romankiewicz & Doevenspeck, 2015), reinforcing the finding that climate and environmental factors operate through more traditional drivers, such as the desire to escape entrenched poverty in source areas. Thus, in some senses, climate change may be blamed for migration that has roots in deep historical inequalities, historic emissions, and power imbalances at national and international levels, revealing that the burden of adaptation falls disproportionately on the poor (Gonzalez, 2020; Ribot et al., 2020; Bettini & Goli, 2016).

While most climate mobility is anticipated to be internal (DFID, 2009), statistical methods have been used to calculate the number of international migrants and asylum seekers who may have moved owing to climate impacts, and even to project these numbers in the future based on climate scenarios (Abel et al., 2019; Feng et al., 2010; Missirian & Schlenker, 2017). These efforts have been criticized either on methodological grounds (Auffhammer & Vincent, 2012) or due to the many assumptions and uncertainties involved. The facts suggest that international migration stocks have remained relatively stable at 3.5% of global population (UNPD, 2019) and that climate change is not a primary driver (Ribot et al., 2020; Rigaud et al., 2018). Yet, the number of displaced persons stands at a record high (UNHCR, 2020), and climate change has been identified as a threat-multiplier that can make already difficult situations even worse (e.g., Lake Chad Basin, Horn of Africa) (Dalby, 2018). Even a modest increase in refugee numbers threatens to overwhelm an international protection system already at the breaking point (Aleinikoff & Zamore, 2019; Katz, 2020).

The discourse on climate mobility has shifted from framing migration as a negative consequence of climate change impacts to describing it as a form of human adaptation (McLeman & Smit, 2006; Vinke et al., 2020), though the “migration as adaptation” framing has been criticized as a reincarnation of equally rosy portrayals of migration as a necessary feature of economic development (Bettini & Goli, 2016). The debate on migration as adaptation has been extended as scholars consider the translocal dimensions of adaptation, which understands that migrants are often connected to multiple places, including origin and destination areas, and potentially other diaspora communities (McMichael et al., 2021), all of which has been facilitated by the Internet and social media.

From a development perspective, migration as adaptation serves a number of purposes. First, migration of individuals or households to less risky or more suitable environments can reduce exposure to climate hazards (de Sherbinin et al., 2012). Second, at the household-level, migration of one or more individuals can be part of a livelihood diversification and risk-reduction strategy, whereby remittances from household members in destination areas help to support the economic unit and smooth out household consumption during times of distress (Massey et al., 1993). Third, migration can increase household assets in source areas and thereby resilience to climate change. Fourth, migration can reduce the number of household members to support and thereby increase food security for those who remain behind (Rain, 2018). Lastly, returning migrants and migrant associations can bring new skills and knowledge to their communities and countries of origin (“brain gain”).

Migration can also be maladaptive. For example, migrants can move into areas of greater risk (e.g., informal settlements at risk of flooding and landslides); migration can deprive communities of labor (“brain drain”), and can contribute to the decline of rural communities, making them less resilient to climate impacts (Black et al., 2011). Too often, migrants experience serious risk of injury, exploitation, and death on their journeys, especially in the context of large mixed movements of refugees and other “survival migrants” (Betts, 2010), as exemplified by recent large movements from Syria, Myanmar, Venezuela, and Guatemala. Finally, forced displacement, while serving as a survival mechanism, especially in the context of sudden onset disasters, often undermines development gains and leaves people in situations of limbo.

The precise balance of benefits and risks of migration varies significantly based on the degree to which the decision to move is voluntary and well-informed, the profiles of those who move (age, health, assets, skills etc.), the context of their journeys, conditions in destination areas, and the ability to stay connected with people and places left behind (UNDP, 2009). Various policy frameworks address both the potential opportunities and risks that come with migration and displacement in the context of climate hazards and disasters (for a comprehensive overview see: IOM, 2018b). However, none establishes binding rules and few provide detailed policy guidance.

The Cancun Adaptation Framework (2010) invites States to coordinate and cooperate on “climate induced displacement, migration and planned relocation.” Meanwhile, displacement is framed as a form of “loss and damage” under the United Nations Framework Convention on Climate Change’s Warsaw International Mechanism on Loss and Damage from 2013. COP 21 in 2015 created the Taskforce on Displacement, an advisory body tasked with developing recommendations for integrated approaches to “avert, minimize and address displacement related to the adverse impacts of climate change” (UNFCCC, 2015: paragraph 49).

The Sendai Framework for Disaster Risk Reduction (2015) conceptualizes population movements as both a source of risk and a contributor to strengthening the resilience of people and communities. It further recognizes that migrants, with their knowledge, skills, and capacities, can contribute to the design and implementation of disaster risk reduction measures.

The 2030 Agenda for Sustainable Development calls for “leaving no one behind,” explicitly recognizing the need to empower people who are vulnerable, including refugees, internally displaced persons and migrants and to enhance the resilience of refugee hosting communities. The Sustainable Development Goals (SDGs) feature several migration-related targets, including target 10.7 to “facilitate orderly, safe, and responsible migration and mobility of people, including through implementation of planned and well-managed migration policies,” which is framed as a contribution to the goal of reducing inequality.

The Global Compact for Safe, Orderly and Regular Migration (shortly Global Compact for Migration, GCM, 2018), the first comprehensive—but non-binding—international agreement to spell out how states ought to manage international migration, is built on the idea that migration should be a choice, not a necessity; involuntary movements should be reduced and prevented, as should irregular migration that undermines the rule of law and leaves migrants vulnerable to abuse and exploitation.

The GCM outlines a comprehensive set of policy recommendations for managing movements induced by “natural disasters, the adverse effects of climate change and environmental degradation” (UNGA, 2018a: 10/36), including actions to minimize adverse drivers of migration by strengthening resilience and adaptation strategies, integrating displacement into disaster preparedness, and undertaking joint contingency planning (GCM Objective 2), as well as actions to enhance pathways for regular migration, including through both temporary and permanent legal avenues for climate migration, where necessary (GCM Objective 5). Table 1 provides a more detailed overview of relevant GCM provisions.

**Table 1:** Global compact for migration language on climate migration

Field of action	GCM language
(1) Analysis and information- sharing	Strengthen joint analysis and sharing of information to better map, understand, predict, and address migration movements, such as those that may result from sudden-onset and slow-onset natural disasters, the adverse effects of climate change, environmental degradation, and other precarious situations, while ensuring the effective respect, protection, and fulfillment of the human rights of all migrants
(2) Adaptation and resilience strategies	Develop adaptation and resilience strategies to sudden-onset and slow-onset natural disasters, the adverse effects of climate change, and environmental degradation, such as desertification, land degradation, drought, and sea level rise, taking into account the potential implications on migration, while recognizing that adaptation in the country of origin is a priority
(3) Disaster preparedness, early warning and contingency planning	Integrate displacement considerations into disaster preparedness strategies and promote cooperation with neighboring and other relevant countries to prepare for early warning, contingency planning, stockpiling, coordination mechanisms, evacuation planning, reception and assistance arrangements, and public information
(4) Reception and humanitarian assistance	Harmonize and develop approaches and mechanisms at subregional and regional levels to address the vulnerabilities of persons affected by sudden-onset and slow-onset natural disasters, by ensuring they have access to humanitarian assistance that meets their essential needs with full respect for their rights wherever they are, and by promoting sustainable outcomes that increase resilience and self-reliance, taking into account the capacities of all countries involved
(5) Admission and stay	Develop or build on existing national and regional practices for admission and stay of appropriate duration based on compassionate, humanitarian or other considerations for migrants compelled to leave their countries of origin, due to sudden-onset natural disasters and other precarious situations, such as by providing humanitarian visas, private sponsorships, access to education for children, and temporary work permits, while adaptation in or return to their country of origin is not possible
(6) Relocation	Cooperate to identify, develop, and strengthen solutions for migrants compelled to leave their countries of origin due to slow-onset natural disasters, the adverse effects of climate change, and environmental degradation, such as desertification, land degradation, drought, and sea level rise, including by devising planned relocation and visa options, in cases where adaptation in or return to their country of origin is not possible
(7) International policy development	Develop coherent approaches to address the challenges of migration movements in the context of sudden-onset and slow-onset natural disasters, including by taking into consideration relevant recommendations from State-led consultative processes, such as the Agenda for the Protection of Cross-Border Displaced Persons in the Context of Disasters and Climate Change, and the Platform on Disaster Displacement

The GCM includes a detailed set of policy options on climate change and migration, largely because States have been reluctant to address this issue in the context of the existing refugee protection regime established in the 1951 Refugee Convention and its 1967 Protocol, which do not recognize people displaced due to the impacts of climate or environmental changes.

The Global Compact on Refugees (GCR), developed and adopted in parallel to the GCM in 2018, acknowledges that “climate, environmental degradation and natural disasters increasingly interact with the drivers of refugee movements,” but does not recognize them as drivers in their own right (GCR, 2018: para 8). According to UNHCR, multiple provisions within the GCR can however be interpreted to suggest its applicability to situations of cross-border displacement in the context of disasters and environmental degradation (UNHCR, 2019). Some regional refugee protection frameworks, such as the Organization of African Unity Refugee Convention and the Cartagena Declaration in the Americas, as well as the Guiding Principles on Internal Displacement (1998) include provisions that explicitly or implicitly extend protection to those moving due to disasters or environmental causes, and States have used them to that effect (Adeola, 2020; Weerasinghe, 2018).

Much of the policy agenda outlined in the GCM builds on the work of the Nansen Initiative, an informal process initiated by the Governments of Norway and Switzerland in 2012, and its successor, the Platform for Disaster Displacement (PDD, 2021). In 2015, 109 countries endorsed the Agenda for the Protection of Cross-Border Displaced Persons in the Context of Disasters and Climate Change, a guidance document that was developed by the Nansen Initiative through a series of consultations with governments and other stakeholders. It covers preparedness before displacement occurs, protection and assistance during displacement, and transition to solutions in the aftermath of a disaster. The Platform for Disaster Displacement works to promote the implementation of the Protection Agenda and serves as a center of gravity convening governments and connecting them with researchers, international and non-governmental organizations active in the fields of climate and migration.

Given the number of different frameworks that govern the nexus of climate change, migration, and displacement, inevitable challenges of policy coherence arise both within the climate and the migration sectors, as well as across them. For instance, climate policy may see migration as a means to support adaptation, yet cross-border movements of people are subject to national and regional migration policies that generally do not conform to climate policy objectives and may be more or less guided by the patchwork of international frameworks that govern migration, from the GCM to the International Convention on the Protection of the Rights of All Migrant Workers and Members of Their Families (1990), the Protocols on Smuggling of Migrants and Trafficking in Persons (2000), and the General Agreement on Trade in Services (WTO, 1995, Mode 4 on the movement of natural persons). Whole-of-government and multi-sector approaches will be needed to address the complexity of the climate-mobility nexus and avoid goal conflicts.

### The role of development cooperation in addressing climate mobility

The field of development cooperation has undergone an important transformation in recent years. The notion of development has evolved, from a narrow focus on economic growth and poverty reduction to a more multifaceted pursuit and assessment of societal progress, reflected in the SDG combination of social, economic, environmental, and good governance objectives. At the same time, new actors and instruments of cooperation have emerged (Klingebiel, 2014). Development cooperation is increasingly understood to be more than aid, or the transfer of financial resources from rich to poor countries. While financing continues to be an important part of development cooperation, other measures, such as capacity development and policy change, have emerged as important levers (Alsonso & Glennie, 2015). This is especially true in the areas of climate change and migration, where what developed countries do at home—in terms of carbon emission reductions and visa and integration policies—may have greater effects on developing countries than any aid they give.

Alonso and Glennie (2015:5) define development cooperation as “Activity that aims explicitly to support national or international development priorities, is not driven by profit, discriminates in favor of developing countries, and is based on cooperative relationships that seek to enhance developing country ownership.” There can be tension between principles for good development cooperation, such as the commitment to country ownership enshrined in the Busan Partnership for Effective Development Cooperation (2011), and the use of development assistance to address climate and migration policy goals. Aid may be targeted away from the poorest countries and people and subject to political pressures, e.g., to deter migrants from coming to rich countries and induce people to remain in their regions of origin (Hooper & Newland, 2018). Despite pledges for parity in spending on climate mitigation and adaptation, and an overall increase in climate finance between 2013 and 2017, only about 20% of bilateral and 27% of multilateral climate finance went to adaptation programmes (OECD, 2018). Public funding for adaptation totaled $22 billion in 2016, a fraction of the $140–300 billion per year that UNEP estimates will be needed by 2030 (Chan & Amerasinghe, 2018).

Our discussion of the role of development cooperation actors is based on practices of traditional donors channeled through bilateral or multilateral development agencies. A search on “climate AND migration” of the AidData database, which draws on Organization for Economic Cooperation and Development (OECD) records, yields a total of 222 projects (out of a total of almost 1.7 million entries) by 19 donor agencies between 2000 and 2014, ranging in investment from $263.5 million by the World Bank, $17 m by the USAID, $12 m by the former Canadian International Development Agency, $5 m by the Global Environmental Facility, and $2.6 m by German Development Cooperation (GIZ). The top program countries by funding volume are India, followed by China, Afghanistan, Senegal, Thailand, Zimbabwe, Haiti, and the Federated States of Micronesia. Over two-thirds of the recorded interventions are coded as multi-sector, followed by those categorized as disaster prevention and preparedness, environmental protection, basic health, and conflict prevention and resolution. The majority of the projects are research related, designed to generate knowledge on the links between climate and migration in regions thought to be particularly susceptible to climate change impacts. Another focus area is support for livelihoods in climate-impacted areas and for the participation of affected populations in policy development.

Development interventions tend to be place-based rather than focused on a particular group of movers. As such, they cut across the whole spectrum of types of movements, including internally displaced persons and refugees, return migrants, pastoralists or transhumance, seasonal and labor migration, and rural to urban migration. The bulk of interventions is concerned with internal, rather than international movements related to climate impacts (Rigaud et al., 2018; World Bank, 2019; Yonetani, 2018).

Development cooperation actors’ approach to climate mobility can be grouped into four categories:

1. Adaptation and resilience-building in place

A first entry point for development cooperation actors is to use a displacement prevention lens on their adaptation and resilience-building programs. As the World Bank observes: “Even with expected out-migration, many climate-vulnerable areas will still need to support significant numbers of people. This increases the need for development strategies to support people to adapt locally or ‘stay in place’ in areas where it makes sense to do so” (World Bank, 2019: 29). A recent USAID report on climate mobility similarly focuses on the need to invest in disaster risk reduction and improved agricultural methods, though many of its recommendations (“improve livestock management,” “reduce deforestation,” and “improve housing construction”) remain at a high level (USAID, 2021).

Poverty reduction and social protection programs targeted at rural areas can help to increase adaptive capacity to climate change (Johnson et al., 2013). While countries may pursue different pathways to adaptation (Chapman et al., 2016), frequent components of successful local adaptation strategies include investing in climate-smart infrastructure, diversifying income generating activities, building more responsive financial protection systems for vulnerable groups, and educating and empowering women. Likewise programs dedicated to disaster risk reduction (DRR) may enhance local resilience and reduce the need to migrate (UNDRR, 2019). Indeed, there is often a significant overlap between climate adaptation and DRR approaches, both of which aim for vulnerability reduction (Cardona et al., 2012). In practice, this often translates into a focus on issues such as natural resource management, land use planning, and the rollout of risk assessments and early warning systems. Yet, the time horizon for DRR interventions is usually more short term (focused on immediate risks) and, institutionally, DRR is typically the domain of civil defense or emergency response agencies, while climate change falls under the purview of ministries of the environment and adaptation falls under multiple line ministries (depending on the sector). Among the projects captured in the AidData dashboard, the USA features prominently as a donor with a multi-country investment in supporting capacity building for disaster preparedness in cooperation with the International Organization for Migration (IOM).

While much adaptation programming is focused on livelihoods, that alone seems insufficient when considering probable causes affecting people’s decisions to migrate: Gamso and Yuldashev (2017) find that government inefficiency, unaccountability, and irresponsibility are important factors in encouraging outward migration, particularly among the poor. Governance aid, geared to strengthen political institutions, is accompanied by reductions in the emigration rates of developing countries, whereas social and economic aid has no discernible relationship to emigration. These findings are confirmed by UNDP’s *Scaling Fences* report (UNDP, 2019), which observes that migration from Africa is driven by a sense of hopelessness rather than poverty or unemployment. Where people lack a say in how to adapt to climate variability and changes in the environment, there is a risk that planning and development choices will exacerbate their vulnerability (De Haas, 2020), for instance when tree planting for carbon removal undermines other forms of land use that support people’s food security (Lee, 2019).

Going forward, ensuring financing for adaptation will be a critical consideration. Supporting partner countries to ensure that development strategies and proposals systematically include a climate risk lens could help stretch scarce adaptation funds (Chan & Amerasinghe, 2018). Innovative financing mechanisms such as debt swaps could provide disaster-prone countries that are highly indebted with the financial liquidity to invest in resilience and climate adaptation (Commonwealth Secretariat, 2015; Jubilee Debt Campaign, 2018). Remittances can play a key role in household and macro-level resilience. Creating financial products such as micro-credit, livestock insurance, and diaspora bonds could support countries in managing the high costs associated with disaster shocks, strengthen preparedness, and facilitate timely action (Plaza, 2019). Finally, climate index insurance is being tested in many regions to help poor farmers weather climate shocks (Hellmuth et al., 2009).

(2) Facilitating mobility as an integral part of climate adaptation

Just as it is important to reduce the need for people to move under distress, some development interventions recognize the risk of trapped populations who lack the financial and social capital to move out of harm’s way (Foresight, 2011). The United Kingdom’s Department for International Development (former DFID—now UK AID), for instance, explored how the use of cash transfers could enable households and communities to choose migration as an adaptation strategy (see AidData database entry ID#: 906,000,415,839). An internal World Bank review suggests that where there is no credible long-term pathway to viable livelihoods, well-planned migration to more conducive areas can be a successful strategy, including by providing people with the resources they may need to be able to move when “adaptation in place” has reached its limits (World Bank, 2019).

There seem to be few projects proactively supporting people’s use of migration as a means of adapting to climate change impacts. This may be due to a sedentary bias in national development and adaptation plans, where rural–urban migration is often conceived as putting unsustainable pressure on urban infrastructure and services (Black & Sward, 2009; IOM, 2018a; Sward & Codjoe, 2012). Reviewing 82 national and regional Disaster Risk Reduction Strategies, Yonetani (2018: 12) finds that “voluntary migration’s potentially positive contribution to resilience is seldom recognized. Few provisions were found to support (labor) migration as a form of adaptation or a coping strategy to avoid disaster.” However, IOM also observes that national strategies such as National Adaptation Plans, Intended Nationally Determined Contributions, Nationally Determined Contributions, and National Communications exhibit an increasing policy awareness of human mobility being driven by, and an adaptive response to, the adverse impacts of climate change (IOM, 2018a).

One example of a regional approach to climate-related mobility is the World Bank’s Regional Sahel Pastoralism Support Project for the countries of Burkina Faso, Chad, Mali, Mauritania, Niger, and Senegal (World Bank, 2019). The project supports trans-boundary migration as an adaptation strategy for pastoralists threatened by climate change-induced droughts and conflict by improving the sub-regional infrastructure (e.g., migration corridors, markets for regional trade in livestock products, and shared water points) for migrating pastoralists. This included the building of migration corridors, support for regional trade in livestock products, and the establishment of shared water points. Moreover, the project fostered regional collaboration and coordination to better manage shocks affecting livestock, such as drought and disease. There is growing interest in the potential of regional solutions and regional free movement frameworks (such as those enshrined under protocols of the Economic Community of West African States and the East African Community, for instance). The United Nations Migration Multi-Partner Trust Fund, which supports the implementation of the GCM (MPTF, 2021), supports programs in the Pacific Islands and East Africa that seek to facilitate labor mobility and regular migration in the contexts of disasters and climate change. The European Union-supported Pacific Climate Change and Migration project sought to increase the protection of individuals and communities vulnerable to climate change displacement and migration through targeted national and regional policies. It also pursued increased labor mobility opportunities for Pacific Islanders, through well-managed labor migration schemes (see Oakes et al., 2016). The New Zealand Government has been deliberating options for managing climate related mobility in the face of rising sea levels across the Pacific Islands region (New Zealand MFAT, 2018).

- (3)Moving people out of harm’s way: planned relocation or resettlement

There is clear recognition that moving people away from harm is an essential part of saving lives in an emergency. The most common reference to human mobility in DRR strategies is in the context of planned relocation preparedness (Yonetani, 2018). Country strategies such as National Adaptation Programmes of Action and national DRR strategies also recognize the need to relocate or resettle populations where their lives and livelihoods are threatened due to sea-level rise, flooding and landslides (Sward & Codjoe, 2012). International Organization for Migration’s mapping of National Adaptation Plans, Intended Nationally Determined Contributions, Nationally Determined Contributions, and National Communications find references to relocation for instance in country submissions by Uruguay, Canada, Cuba, Fiji, Malta, Rwanda, and Samoa (IOM, 2018a). Relocation and resettlement are considered both a preventative measure for people living in situations of increasing disaster and displacement risk, as well as a post-disaster rehabilitation and recovery measure for displaced people unable to return home (de Sherbinin et al., 2011; Ferris, 2014; Yonetani, 2018).

Development cooperation actors’ involvement in planned relocation processes is trailed by a rather “dark history” of development-related relocations, including large numbers of people affected, lasting negative effects such as impoverishment for the displaced, and a disproportionate impact on indigenous and minority communities (Robinson, 2003). Oliver-Smith and de Sherbinin (2014) observe that disaster-related resettlement interventions have had a more successful track-record than development-related resettlements, such as those associated with large dams. However, they caution that resettlement should always be an option of last resort. Indeed, much of the literature on relocations insists on the need for clear legal and policy frameworks, such as the Guiding Principles on Internal Displacement, to guide such efforts (e.g., UNHCR/Brookings-LSE/IMI 2015; Ferris, 2014; de Sherbinin et al., 2011). Robinson (2003) stresses the importance of incorporating both an “assessment of risks” and a “recognition of rights” to avoid poor outcomes.

Connell and Coelho (2018) observe that as more countries are developing policies to address climate related relocation challenges, the involvement of multiple ministries and levels of government is critical. Indeed, important elements to consider in the process of relocation, such as access to land, livelihoods, and services in relocation areas and questions of financing and compensation (Chun, 2015; Koskinen-Lewis et al., 2016) call for the involvement of planning and development authorities. The World Bank has begun implementing planned relocation projects under its West Africa Coastal Adaptation project in Sao Tome and Principe and Saint-Louis, Senegal (World Bank, 2019). The Government of Fiji developed its National Relocation Guidelines to assist communities affected by sudden and slow-onset processes, with support from German Agency for International Cooperation (GIZ, 2017).

- (4) Managing displacement impacts

As noted above, development cooperation actors have gained an increasing role in responding to large-scale displacement situations in recent years. Recent bilateral compacts between the European Union and Jordan (Grawert, 2019) and several international partners and Ethiopia (Fanuel, 2017) have sought to entice host governments to grant refugees from neighboring countries rights, such as access to the labor market, in exchange for development financing and trade concessions. The World Bank created its Global Concessional Financing Facility^3^ and the refugee window under The International Development Association (IDA) 18 to provide concessional financing to low- and middle-income countries that host large numbers of refugees. Country programs that are specifically targeting refugee receiving areas combine place-based interventions that benefit the entire local population (e.g., infrastructure development, improvements in public services) with interventions that focus on the short-term needs of the displaced (e.g., specialized education, psychosocial support, vocational training) (World Bank, 2019).

Where displaced populations settle in hazard-prone areas, they may be vulnerable to climate and disaster impacts, including the risk of consecutive displacement. For instance, rapidly growing megacities in river deltas such as Dhaka and Lagos are preferred migration destinations largely due to promising labor opportunities (Black et al., 2011), even though migrants in crowded urban environments often report a decline in wellbeing (Ayeb-Karlsson, 2020). Leveraging existing social (community, migration, etc.) networks could be an important mechanism in this context, since the existence of social institutions (churches, mosques, savings associations, and mutual aid) has been found to increase resilience (Adger et al., 2011; Trzaska et al., 2017).

The International Organization for Migration is working to ensure that migrants and internally displaced persons are part of DRR planning and responses. The Migrants in Countries in Crisis initiative, hosted by IOM, offers guidelines and a repository of good practices for the inclusion of migrants in disaster and emergency preparedness, response, and recovery. IOM also offers countries support for tracking displacement to inform their disaster response (IOM, 2017).

Other interventions seek to address the environmental impacts of displaced populations in host communities and to make the response to displacement more sustainable, for instance, through infrastructure updates, improved waste management in cities (European Bank for Reconstruction and Development in Amman), or the use of renewable energy solutions in refugee camps (e.g., World Bank, 2019).

We have reviewed development cooperation actors’ engagement on climate mobility and found four categories of interventions: enabling people to stay through in situ adaptation measures, facilitating migration to support adaptation, facilitating planned relocations and resettlement to protect people from harm, and supporting destination areas in the reception of migrants.

Going forward we see a need for development cooperation actors to engage directly with affected communities and levels of government to co-design interventions that respond to their needs and priorities and assess what works.

## Future prospects and policy implications

The Covid-19 pandemic has been a large negative shock for development, throwing millions of people into poverty and leaving developing country governments with limited resources to support the poor and stimulate their economies without risking debt crises (UN/DESA, 2020). It has also resulted in widespread unemployment (and even “internal deportation” in India) of urban migrant laborers (Khanna, 2020), and a hardening of borders and more restrictive travel policies that have hindered international movements (O’Brien & Eger, 2020). As such, the Covid-19 pandemic could further deepen inequalities within and across countries and undermine the resilience of vulnerable people and nations to cope with the larger climate crisis.

The SDGs recognize that safe and orderly migration can minimize inequalities within and across countries. The question is how migration can happen in a safe and orderly manner under conditions of increasingly severe climate hazards. Governments at various levels, development agencies, and international and non-governmental organizations are already struggling to manage migration and displacement in a manner that protects rights, reduces vulnerability and creates development opportunities. Prospects for scaling the ability to manage movements in line with need—whether for facilitated mobility or decent reception and long-term inclusion of the displaced—seem severely constrained by a lack of proactive adaptation and urban planning in the case of internal movements, and the political realities of rising nationalism and xenophobia for international migration. In a world shaped by the pandemic, the possible introduction of new travel requirements such as vaccine certificates will likely make international movements even more selective, while fuelling the underground business of smugglers.

In contrast to the Covid-19 pandemic, no vaccine for climate change is envisioned that would reverse global warming and its negative impacts on social, economic, and ecological systems. Ahead of COP-26 in Glasgow and with the arrival of the new United States (US) administration, there is however some hope for renewed political momentum around the need for ambitious climate action. Indeed, the US administration is showing an appetite for leadership not just on climate change, but also on migration. In a recent Executive Order on refugee resettlement and planning for the impacts of climate change on migration, President of the USA, Joseph R. Biden requested his administration to produce a report on “climate change and its impact on migration, including forced migration, internal displacement, and planned relocation” within 180 days (White House, 2021, Sec.6). Besides consideration of the international security implications, the report is to include “options for protection and resettlement of individuals displaced directly or indirectly from climate change” and “proposals for how these findings should affect use of United States foreign assistance to mitigate the negative impacts of climate change” (White House, 2021, Sec.6).

Arguably, the report (White House, 2021) presents a number of important openings to (a) mobilize whole-of-government and whole-of-society engagement in the report drafting process, reflective of the many sectors and actors that can contribute to building out the climate mobility “toolbox”; (b) adopt a deliberate, developmental approach to climate mobility that counterbalances security narratives by recognizing the positive contributions of migrants and refugees to the societies they leave and join; and (c) articulate how US political leadership and the strategic use of its external cooperation tools can be leveraged to support climate adaptation and resilience-building in the most vulnerable countries, cities and communities; the development of cooperative arrangements for facilitated mobility; and the permanent resettlement of displaced people in the US. In seizing this opportunity, the US can set an example for others and rally them. It can also assert its influence in multilateral development banks and agencies.

For those banks and agencies, continued investment in improving the evidence base for interventions, including through direct engagement with affected communities, will be critical. We conclude by outlining three areas for further exploration in this regard:

1. Dynamics in climate hotspots: Much research has focused on the most vulnerable areas, or “hot spots” of potential out-migration. This includes low-lying regions and islands with high population density and coastal agricultural areas (e.g., river deltas). However, beyond understanding exposure to the physical impacts of climatic changes, it will be increasingly important to understand feedback loops, i.e., secondary effects in economic and social systems, and how those are affecting people’s vulnerability and propensity to move. This also requires improved monitoring and evaluation of the impacts (and potentially unintended consequences) of development and adaptation policy and programs. It will also be critical to ensure that climate information is generated in consultation with, and available to, the communities that need it most to make informed decisions regarding adaptation choices.
2. Development in climate mobility destinations: Development cooperation needs to focus attention on the current and potential future destination areas of climate mobility, including the impact of mobility on the latter’s adaptation pathways. To support positive outcomes for sustainable development, it will be necessary to channel resources towards, and work directly with, local authorities and communities to help them anticipate and respond to climate mobility pressures and opportunities. Poor and marginalized populations residing in slums or squatter settlements that are particularly prone to environmental hazards must be a key consideration (de Sherbinin et al., 2007; Gemenne et al., 2020). Groups like the Mayors Migration Council and the C40 Cities are working together to support cities in assessing and managing the interactions of climate change, migration and inclusion in their jurisdictions.
3. Leveraging transnational migrant ties for development: There is scope for further exploring and developing programming around the role of translocal and transnational ties in building resilience (Greiner et al., 2015; Farbotko, 2020). This includes, but is not limited to, supporting the role of remittances in building household and macro-level resilience and developing financial products that could be used to enhance their impact (Plaza, 2019).

Climate mobility, while raising legal and operational challenges, requires political solutions to be negotiated at various scales—be it cities coming together in regional coalitions to do joint adaptation planning (Shi, 2017), governments negotiating bilateral agreements to facilitate resettlement (Amakrane, 2021), or regional bodies incorporating climate mobility as part of regional free movement agreements (Wood, 2019). Development cooperation actors can support such efforts and use their convening power to facilitate joint analysis, planning and implementation of strategies across multiple levels of governance and sectors of policy making at national level, and to support knowledge-sharing and coalition-building among similarly affected countries and communities across borders. Their quest for eliminating extreme poverty, reducing inequalities and generating prosperity ultimately depends on it.

## Footnotes

1 In line with the broad development notion underpinning the Sustainable Development Goals, this includes specialized entities such as the International Organization for Migration, for instance.

2 There are ongoing discussions about expanding the definition of refugees to include those affected by climate impacts (Gonzalez, 2020), but this is beyond the scope of this article. It is important to recognize that terminology is not solely a matter of semantics. Definitions and legal categorizations do matter to development cooperation, since it may affect who qualifies for assistance.

3 It provides development support on concessional terms to middle-income countries impacted by refugee crises across the world.
